# Foxa1 and Foxa2 in thymic epithelial cells (TEC) regulate medullary TEC and regulatory T-cell maturation

**DOI:** 10.1016/j.jaut.2018.07.009

**Published:** 2018-09

**Authors:** Ching-In Lau, Diana C. Yánez, Anisha Solanki, Eleftheria Papaioannou, José Ignacio Saldaña, Tessa Crompton

**Affiliations:** a UCL Great Ormond Street Institute of Child Health, 30 Guilford Street, London WC1N 1EH, UK; b School of Health, Sport and Bioscience, University of East London, Water Lane, London E15 4LZ, UK

**Keywords:** Foxa1, Foxa2, T-cell development, TEC, Thymus, Treg

## Abstract

The Foxa1 and Foxa2 transcription factors are essential for mouse development. Here we show that they are expressed in thymic epithelial cells (TEC) where they regulate TEC development and function, with important consequences for T-cell development. TEC are essential for T-cell differentiation, lineage decisions and repertoire selection. Conditional deletion of *Foxa1* and *Foxa2* from murine TEC led to a smaller thymus with a greater proportion of TEC and a greater ratio of medullary to cortical TEC. Cell-surface MHCI expression was increased on cortical TEC in the conditional Foxa1Foxa2 knockout thymus, and MHCII expression was reduced on both cortical and medullary TEC populations. These changes in TEC differentiation and MHC expression led to a significant reduction in thymocyte numbers, reduced positive selection of CD4^**+**^CD8^**+**^ cells to the CD4 lineage, and increased CD8 cell differentiation. Conditional deletion of *Foxa1* and *Foxa2* from TEC also caused an increase in the medullary TEC population, and increased expression of Aire, but lower cell surface MHCII expression on Aire-expressing mTEC, and increased production of regulatory T-cells. Thus, Foxa1 and Foxa2 in TEC promote positive selection of CD4SP T-cells and modulate regulatory T-cell production and activity, of importance to autoimmunity.

## Highlights

• Foxa1 and Foxa2 control thymic epithelial cell differentiation and T-cell selection.
• Foxa1 and Foxa2 in thymic epithelial cells modulate regulatory T-cell production.
• Deletion of *Foxa1* and *Foxa2* from TEC enhances Treg activity.

## 1 Introduction

The thymus is essential for the production of mature T-cells. Signals provided by thymic epithelial cells (TEC) support the development of T-cells and determine the T-cell receptor (TCR) repertoire. Two main populations of TEC, cortical(c) TEC and medullary(m) TEC establish distinct functional microenvironments to facilitate T-cell development. These two TEC populations share a common precursor and have been defined by cell-surface markers and their location in the thymus [1,2].

Cortical TEC (CD45^**-**^EpCam1^**+**^Ly51^**+**^UEA1^**-**^) first provide the Notch ligand DLL4 and IL7 to signal for T-cell fate specification and to support early T-cell progenitor maturation and expansion. The CD4^−^CD8^−^ double negative (DN) thymocyte population then differentiate to become CD4^**+**^CD8^**+**^ double positive (DP) cells. To differentiate further into CD4^**+**^CD8^−^ (CD4 single positive, CD4SP) or CD8^**+**^CD4^−^ (CD8SP) cells, DP thymocytes must express a TCR to interact with major histocompatibility complex (MHC) + peptide complexes on cTEC to induce positive selection. The outcome of positive selection is determined by appropriate strength, duration and timing of TCR signal transduction, and the process ensures that thymocytes that express TCR that interact with MHCII will become CD4SP, whereas cells that express TCR that bind to MHCI will differentiation into CD8SP [2,3]. Thus, expression of cell-surface MHCI and MHCII by cTEC is essential for differentiation to CD8SP and CD4SP respectively.

Following positive selection, the newly produced single positive thymocytes migrate to the medulla, where their interactions with mTEC (CD45^**-**^EpCam1^**+**^Ly51^**-**^UEA1^**+**^) are essential for induction of central tolerance to self. Medullary TEC induce tolerance by providing MHC + peptide ligands to trigger clonal deletion of self-reactive clones, or to drive regulatory T-cell (Treg) maturation. Mature mTEC express the *Aire* gene, which enables expression of Tissue Restricted Antigens (TRA) to induce self-tolerance, and Aire mutation leads to multi-organ autoimmunity [4]. TCR signal strength is believed to be a determinant of clonal deletion and Treg selection, so that CD4SP cells that receive the strongest signals undergo negative selection, but other CD4SP cells that receive relatively high and persistent TCR signalling express CD25 and give rise to Foxp3^**+**^CD25^**+**^CD4^**+**^ Tregs [5].

Foxa1 and Foxa2 are highly conserved and widely co-expressed during murine embryogenesis and in adult tissues, where they function as pioneer transcription factors. Foxa proteins were first discovered by their ability to bind to the promoter of hepatocyte-specific genes and were subsequently shown to regulate metabolic gene expression and liver development [[6], [7], [8]]. In mouse, expression of Foxa2 is required for normal mesoderm and endoderm development as early as E6.5, and constitutive Foxa2 deficiency is embryonic lethal (9–10). Foxa1 is detected at E7.5 in the floorplate, notochord and endoderm, and Foxa1 null mice have defects in the regulation of glucose homeostasis and die shortly after birth due to hypoglycaemia [[9], [10], [11]]. The highly conversed DNA-binding domains among the Foxa proteins and the co-expression of Foxa1 and Foxa2 in various tissues suggested that they play compensatory roles during development and in the regulation of multiple adult tissues [12]. Foxa1 and Foxa2 are co-expressed in the epithelium of many tissues, including lung, gut, pancreas and prostate. Analysis of the impact of individual or combined conditional deletion of Foxa1 and Foxa2 demonstrated that their expression in epithelial cells is important for the development and differentiation of these tissues [[13], [14], [15], [16]]. In the liver, lung and pancreas, conditional deletion of both Foxa1 and Foxa2 resulted in severe tissue-specific defects, whereas conditional ablation of either Foxa gene alone did not interfere with tissue architecture and cell differentiation, demonstrating compensatory and over-lapping functions in these tissues [8,13,17].

Foxa2 is expressed in thymocytes, and a recent study has demonstrated Foxa1 expression in a new subset of Treg that are important for immunosuppression of autoimmune diseases in mouse models [18,19].

Here we show that Foxa1 and Foxa2 are also required for normal TEC differentiation and function, with important consequences for T-cell development and regulatory T-cell selection.

## 2 Methods

### 2.1 Mice

*Foxa1*^flox/flox^*Foxa2*^flox/flox^ mice provided by S-L Ang [20], and Foxn1-cre-transgenic mice by G. Holländer [21], were bred and maintained in individually ventilated cages on C57BL/6 background at University College London under Home Office regulations, and crossed to generate *Foxa1*^flox/flox^*Foxa2*^flox/flox^Foxn1-cre^**+**^ mice (referred to as Foxa1/2Foxn1cKO), using littermate *Foxa1*^flox/flox^*Foxa2*^flox/flox^Foxn1-cre^**-**^ as control.

### 2.2 Genotyping

DNA extraction and PCR analysis were as described [22], using Foxn1-cre primers described [21] and *Foxa1* wild type (WT) and floxed gene: forward 5′CTGTGGATTATGTTCCTGAT3′, reverse 5′GTGTCAGGATGCCTATCTGGT3’; *Foxa2* WT and floxed gene: forward 5′CCCCTGAGTTGGCGGTGGT3′, reverse 5′TTGCTCACGGAAGAGTAGCC3’. PCR conditions were 1 min at 94 °C, 1 min at 58 °C, and 1 min at 72 °C for 35 cycles.

### 2.3 Quantitative RT-PCR

RNA extraction, cDNA synthesis and QRT-PCR were as described [23,24], using *Gapdh* for template quantification and normalisation, and Quantitect primers (Qiagen).

### 2.4 Flow cytometry

Thymocytes and TEC were isolated from postnatal (2–4 week old) mice and stained as described [25,26] using combinations of directly-conjugated antibodies from BDPharmingen, eBioscience and Biolegend, acquired on an Accuri™C6 or LSR-II flow cytometer (Becton Dickinson), and analysed using Flowjo. Data are representative of at least 3 experiments.

### 2.5 T-cell activation

Splenocytes or naïve CD4 cells from spleen were cultured with cRPMI with 0.01 μg/mL of anti-CD3 and anti-CD28 at a concentration of 5 × 10^6^ cells/mL in 96-well plates at 37 °C and 5%CO_2_. Cells were harvested at 24 h and analysed by LSR-II flow cytometer.

### 2.6 T-cell proliferation and Treg suppression assay

T-cells were labelled with CFSE as described [27]. CFSE-labelled T cells (10 × 10^4^) were cultured for 4 days with anti-CD28 (1 μg/mL) and rIL2 (20 ng/mL) in the presence or absence of Tregs in 96-well plate pre-coated with anti-CD3 (5 μg/mL).

### 2.7 Purification of naïve CD4 cells and Treg

Splenocytes were treated with RBC lysis buffer before CD4 cells were purified by EasySep Mouse CD4^**+**^ TCell Isolation Kit (Stemcell Technologies) according to the manufacturer's instructions. To obtain naïve CD4 cells and Tregs, CD4 cells were stained with anti-CD4^Alexa Fluor 700^, anti-CD25^Pecy7^, anti-CD44^eFluor 450^ and anti-CD62L^APC^ and sorted using FACSAria III (BD). For Treg suppression assays, sorted CD4^**+**^CD25^**−**^ were used as responder T cells, and CD4^**+**^CD25^**+**^ cells were used as Tregs. For T-cell activation assay, CD4^**+**^CD25^**−**^CD44^**−**^CD62L^**+**^ cells were obtained and used as naïve CD4 T-cells.

### 2.8 Histology

Thymus, spleens and lymph nodes were isolated and fixed in phosphate-buffered formalin (10% vol/vol), paraffin-embedded, and sectioned for H&E staining, performed by Histopathology, Great Ormond Street Hospital. Pictures were photographed by Zeiss AxioCam digital camera with Zeiss Axioplan (NDU) Microscope, 2.5× Objective lens (Plan-Neofluar/0.075NA) and 10× Objective lens (Plan-Neofluar/0.3NA) and acquired by software AxioVision v4.8 (Zeiss).

### 2.9 Microarray data

Publicly available gene-expression microarray datasets from RNA from purified WT TEC (ArrayExpress accession: E-MEXP-3303) [28] were analysed as described [23].

### 2.10 Statistics

Statistical analysis was performed using unpaired two-tailed t-tests and probabilities considered significant if P < 0.05(*) and P < 0.01(**).

## 3 Results

### 3.1 Deletion of *Foxa1* and *Foxa2* from TEC influences mTEC maturation and MHC expression on cTEC and mTEC

**Fig. 1:**
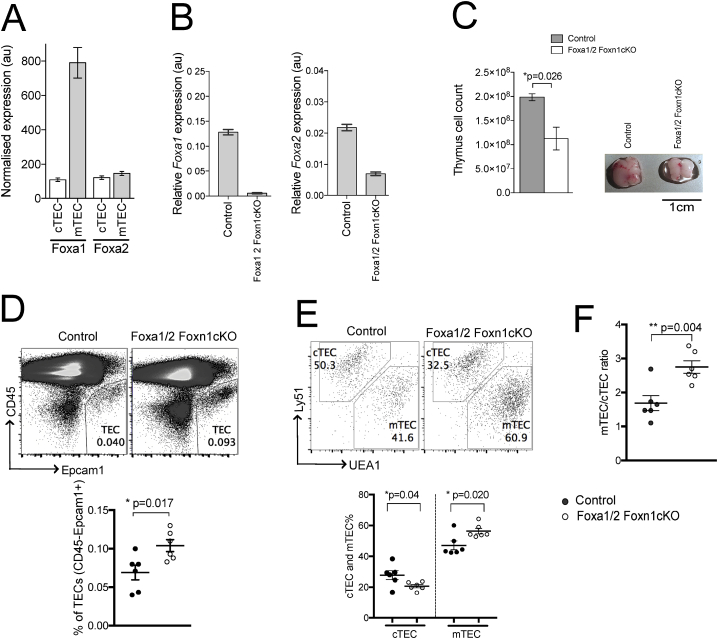
TEC-specific ablation of *Foxa1* and *Foxa2* impairs TEC development. (A) Expression of *Foxa1* and *Foxa2* assessed by microarray in sorted cTEC and mTEC purified from WT thymus. (B) Relative expression of *Foxa1*(left) and *Foxa2*(right) in control and Foxa1/2Foxn1cKO thymus assessed by qRT-PCR. (C) Bar chart shows mean ± SEM cell number of control (n = 6) and Foxa1/2Foxn1cKO (n = 6) thymus. Photograph represents typical thymus from control and Foxa1/2Foxn1cKO mice. (D–F) Flow cytometry analysis of TEC populations isolated from control (n = 6) and Foxa1/2Foxn1cKO (n = 6) thymus. Scatter plots show mean ± SEM. Each point represents thymus from an individual mouse. (D) Dot plot shows anti-CD45 and anti-EpCam1 staining. Gates show percentage of TEC (CD45^**-**^EpCam1^**+**^). Scatter plot shows percentage of TEC in live gate. (E) Dot plots show anti-Ly51 (Ly51^**+**^UEA1^**-**^, cTEC) and UEA1-binding (UEA1^**+**^Ly51^**-**^, mTEC) staining gated on TEC (CD45^**-**^EpCam1^**+**^). Scatter plots show percentage of mTEC and cTEC. (F) Scatter plot shows ratio of mTEC:cTEC.

Analysis of publically available microarray datasets [28] showed that both Foxa1 and Foxa2 are expressed in TEC, with *Foxa1* expression higher in mTEC than cTEC, and *Foxa2* expressed similarly in both TEC subsets (Fig. 1A). Therefore, to investigate their role in TEC development and function, we used the Cre-loxp system to conditionally delete from all TEC [21]. Given their over-lapping and compensatory roles in other tissues, we conditionally deleted both genes, by crossing *Foxa1*^flox/flox^*Foxa2*^flox/flox^ mice with Foxn1-cre mice to excise the floxed alleles from TEC and generate Foxa1/2Foxn1cKO mice. The Foxa1/2Foxn1cKO mice appeared phenotypically normal and we detected no differences in survival or gross differences in the architecture of thymus, spleen and lymph node by haematoxylin and eosin staining between control and Foxa1/2Foxn1cKO (Supp Fig. 1). In the Foxa1/2Foxn1cKO thymus, *Foxa1* was below detection by qRT-PCR, indicating efficient excision from TEC and that overall expression levels of *Foxa1* in other cell types in the thymus is very low. *Foxa2* expression was reduced by ∼60% compared to WT (Fig. 1B), consistent with the finding that *Foxa2* is also expressed by thymocytes [18].

**Fig. 2:**
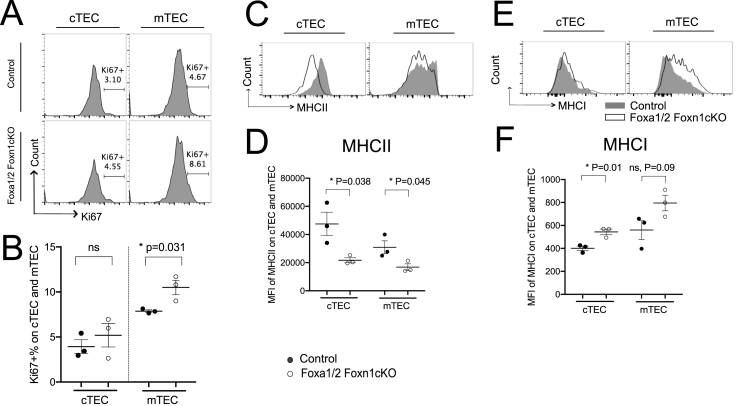
Influence of TEC-specific ablation of *Foxa1* and *Foxa2* on mTEC proliferation and MHC expression on TEC. (A) Histograms show intracellular staining of anti-Ki67 in cTEC and mTEC, giving the percentage of positive cells in the marker shown. (B) Scatter plot shows percentage of Ki67^**+**^ cells, gated on cTEC and mTEC. Histograms show cell surface expression of MHCII (C) and MHCI (E) on cTEC and mTEC. Scatter plots show mean ± SEM MFI of MHCII (D) and MHCI (F) expression on cTEC and mTEC. Each point in scatter plots represents an individual mouse.

The Foxa1/2Foxn1cKO thymus was smaller than control littermate thymus and contained fewer cells (Fig. 1C). We isolated TEC (CD45^**-**^EpCam^**+**^) and analysed cTEC and mTEC populations, by cell surface Ly51 expression and UEA1-binding. The Foxa1/2Foxn1cKO thymus contained a significantly greater proportion of TEC overall (CD45^**-**^EpCam1^**+**^), and of mTEC (UEA1^**+**^Ly51^**-**^), and the ratio of mTEC:cTEC was significantly increased compared to littermate control (Fig. 1D–F). Given this, we investigated the proliferation status of the TEC populations by intracellular-Ki67 staining. The proportion of Ki67^**+**^ mTEC was significantly greater in the Foxa1/2Foxn1cKO thymus compared to control, whereas there was no difference in cTEC, consistent with the expansion of the mTEC population (Fig. 2A–B).

Cell-surface expression of MHC molecules on TEC is essential for T-cell repertoire selection and T-cell lineage decisions [3]. Interestingly, cell-surface MHCII expression was significantly reduced in both cTEC and mTEC populations in Foxa1/2Foxn1cKO compared to control (Fig. 2C–D). The mean fluorescence intensity (MFI) of MHCI, however, was significantly increased in the Foxa1/2Foxn1cKO cTEC population (Fig. 2E–F).

### 3.2 *Foxa1* and *Foxa2* are required for normal positive selection and differentiation to CD4SP

**Fig. 3:**
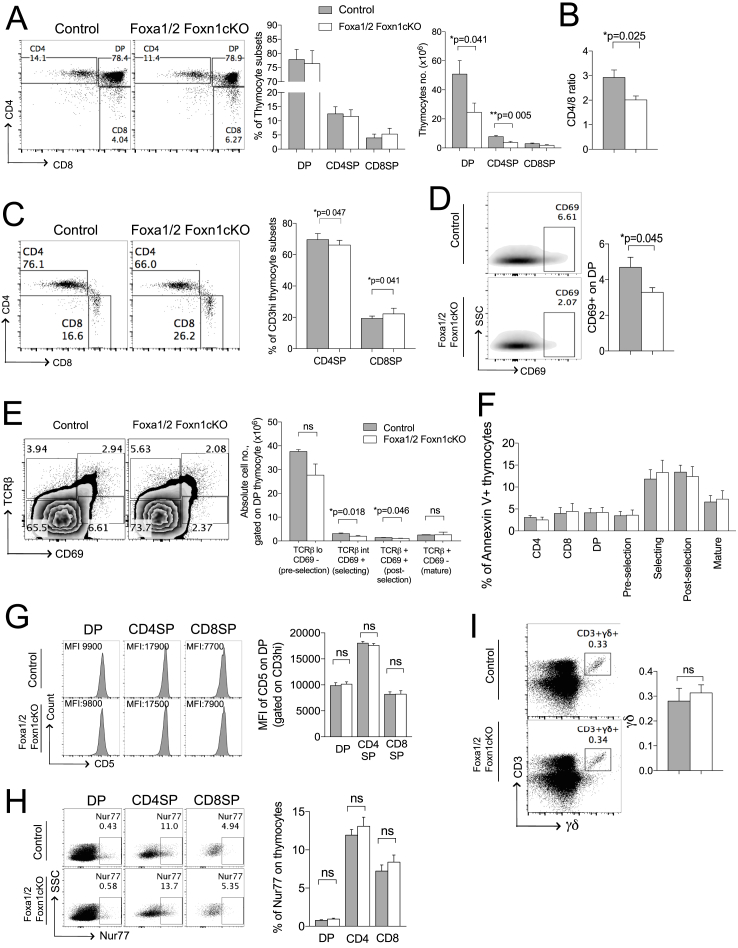
TEC-specific ablation of *Foxa1* and *Foxa2* impairs T-cell development. Flow cytometry analysis of thymocytes. Unless otherwise stated data are from control (n = 6) and Foxa1/2Foxn1cKO (n = 6). (A) Dot plots show CD4 and CD8 expression, giving percentage in region shown. Bar charts show mean ± SEM percentage and number of DP, CD4SP and CD8SP thymocytes. (B) Mean ± SEM ratio of CD4:CD8. (C) Mature CD4SP and CD8SP populations were identified by staining of anti-CD4 against anti-CD8, gated on CD3^hi^ cells, giving percentage in region shown. Bar chart shows mean ± SEM percentage of CD4SP and CD8SP within the CD3^hi^ gate. (D) Expression of CD69 on DP thymocytes, giving percentage in region shown. Bar chart shows mean ± SEM percentage of CD69^**+**^ cells in DP population. (E) Dot plots show TCRβ versus CD69 expression of CD4^+^CD8^+^ thymocyte subsets. Four distinctive DP population were identified, TCRβ^lo^CD69^-^ (pre-selection), TCRβ^int^CD69^+^ (Selecting), TCRβ^hi^CD69^+^ (post-positive selection) and TCRβ^hi^CD69^-^ (mature). Bar chart shows absolute number of each of DP population subsets. (F) Bar chart shows mean ± SEM percentage of Annexin V^**+**^ cells in each thymocyte subsets population for control (n = 4) and Foxa1/2Foxn1cKO (n = 4). (G) Histograms show anti-CD5 staining on thymocytes, gated on CD3^hi^, giving MFI. Bar chart shows mean ± SEM MFI of anti-CD5 staining gated on CD3^hi^ for control (n = 4) and Foxa1/2Foxn1cKO (n = 4) mice. (H) Dot plots show anti-Nur77 intracellular staining on DP, CD4SP and CD8SP thymocytes. Bar chart shows mean ± SEM percentage of anti-Nur77 intracellular staining. (I) CD3 and γδTCR staining in thymus, giving percentage of cells in the CD3^**+**^γδ^**+**^ region shown. Bar chart shows mean ± SEM percentage of γδ T-cells for control (n = 3) and Foxa1/2Foxn1cKO (n = 3).

We then investigated if the changes in TEC development and MHC expression in the Foxa1/2Foxn1cKO thymus influenced T-cell development. We first examined if the levels of cell surface MHC expression on cTEC affected positive selection and CD4/8 lineage choice. The number of DP and CD4SP thymocytes were significantly reduced in the Foxa1/2Foxn1cKO thymus compared to control, and the ratio of CD4SP:CD8SP was decreased (Fig. 3A–B), indicating that the reduction in MHCII expression and increase in MHCI expression in the Foxa1/2Foxn1cKO cTEC population favoured differentiation to CD8SP, and reduced positive selection of CD4SP cells. Gating on CD3^hi^ confirmed that there was a significant reduction in the proportion of mature CD3^hi^CD4SP cells and increase in the proportion of mature CD3^hi^CD8SP cells (Fig. 3C). Furthermore, there was a significant reduction in the proportion of CD69^**+**^ cells in the Foxa1/2Foxn1cKO DP population compared to control, indicating that fewer DP thymocytes were entering the selection process [29]. To dissect further the dynamics of the selection process in the DP population, thymocytes were stained with anti-TCRβ and anti-CD69 to identify four DP thymocyte subsets at different stages of positive selection: TCRβ^lo^CD69^**-**^ (pre-selection), TCRβ^int^CD69^**+**^ (selecting), TCRβ^hi^CD69^**+**^ (post-positive selection) and TCRβ^hi^CD69^-^ (mature) [30]. This analysis revealed that the number of selecting (TCRβ^**-**^CD69^**+**^) and post-positive selection (TCRβ^**+**^CD69^**+**^) DP cells were reduced in Foxa1/2Foxn1cKO mice compared to control (Fig. 3E) confirming that Foxa1 and Foxa2 expression in TEC is required for efficient positive selection. We detected no significant changes in the proportion of cells that stained positive with AnnexinV in any thymocyte population examined, and likewise no differences in levels of cell-surface CD5 expression and intracellular staining against Nur77 in CD3^hi^DP, CD3^hi^CD4SP and CD3^hi^CD8SP between the two genotypes (Fig. 3F–H). As intensity of CD5 and Nur77 expression correlates with TCR signal strength [31], this suggested that after initiation of positive selection, there was no difference in TCR signal strength between genotypes.

Thus, the reduction in positive selection to CD4SP was likely the direct result of a reduction in the proportion of cells that entered positive selection as a consequence of binding MHCII on cTEC, rather than a reduction in TCR signal strength in individual thymocytes, or a thymocyte-intrinsic distortion of TCR signalling as a result of another functional change in TEC [25,[32], [33], [34], [35]]. The development of γδT-cells does not require cTEC for MHC-dependent selection processes, and we found no difference in the proportion of γδT-cells between Foxa1/2Foxn1cKO and control thymus (Fig. 3I).

### 3.3 *Foxa1* and *Foxa2* in TEC modulate regulatory T cell maturation

**Fig. 4:**
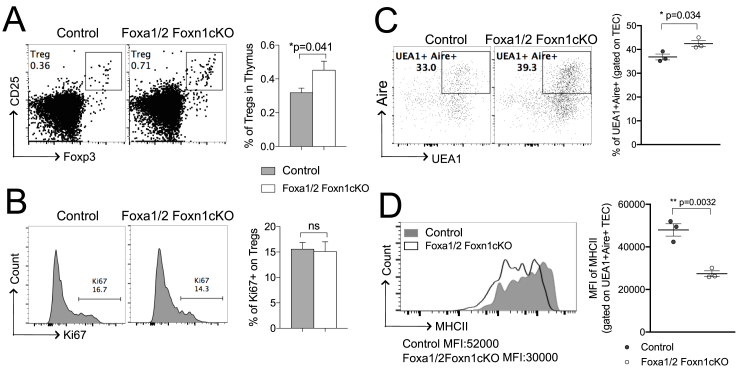
Increased proportion of Treg in Foxa1/2Foxn1cKO thymus. (A) CD25 and intracellular-Foxp3 staining, gated on CD4SP, giving percentage in region shown. Bar chart shows mean ± SEM percentage of Tregs for control (n = 6) and Foxa1/2Foxn1cKO (n = 6). (B) Histograms show intracellular-Ki67 staining, gated on Tregs (CD4^**+**^CD25^**+**^Foxp3^**+**^). Bar chart shows mean ± SEM percentage of Ki67^**+**^ cells in Treg population for control (n = 6) and Foxa1/2Foxn1cKO (n = 6). (C) Dot plots show UEA1-binding against intracellular Aire staining, gated on CD45^**-**^Epcam1^**+**^ (TEC), giving the percentage of Aire^**+**^UEA1^**+**^ mTEC in the region shown. Scatter plots show mean ± SEM percentage of Aire^**+**^UEA1^**+**^ mTEC. (D) Histograms show MHCII expression, gated on Aire^**+**^UEA1^**+**^ mTEC for control (n = 3, grey solid) and Foxa1/2Foxn1cKO (n = 3, black line). Scatter plots show mean ± SEM MFI of MHCII gated on Aire^**+**^UEA1^**+**^ mTEC. Each point represents an individual mouse.

As mTEC were increased in the Foxa1/2Foxn1cKO thymus and mTEC play a primary role in Foxp3^**+**^ Treg selection [36], we investigated Treg populations. The proportion of Tregs in the CD4SP population was significantly increased in Foxa1/2Foxn1cKO compared with control (Fig. 4A), but there was no difference in Ki67 expression in these cells, indicating that the increase was not due to increased Treg proliferation after their selection (Fig. 4B). Treg selection is predominantly Aire-dependent [37,38], so we examined Aire expression in mTEC. Gating on the UEA1^**+**^ (mTEC) population, the percentage of Aire^**+**^ cells was significantly increased in the Foxa1/2Foxn1cKO thymus (Fig. 4C). However, expression of cell-surface MHCII was significantly lower on Foxa1/2Foxn1cKO Aire^**+**^mTEC (Fig. 4D), indicating that although these cells have capacity to express TRA, they would express MHCII + TRA at lower cell-surface density than the control, potentially favouring Treg selection over negative selection.

### 3.4 Influence of conditional deletion of Foxa1 and Foxa2 from TEC on peripheral T-cell population

**Fig. 5:**
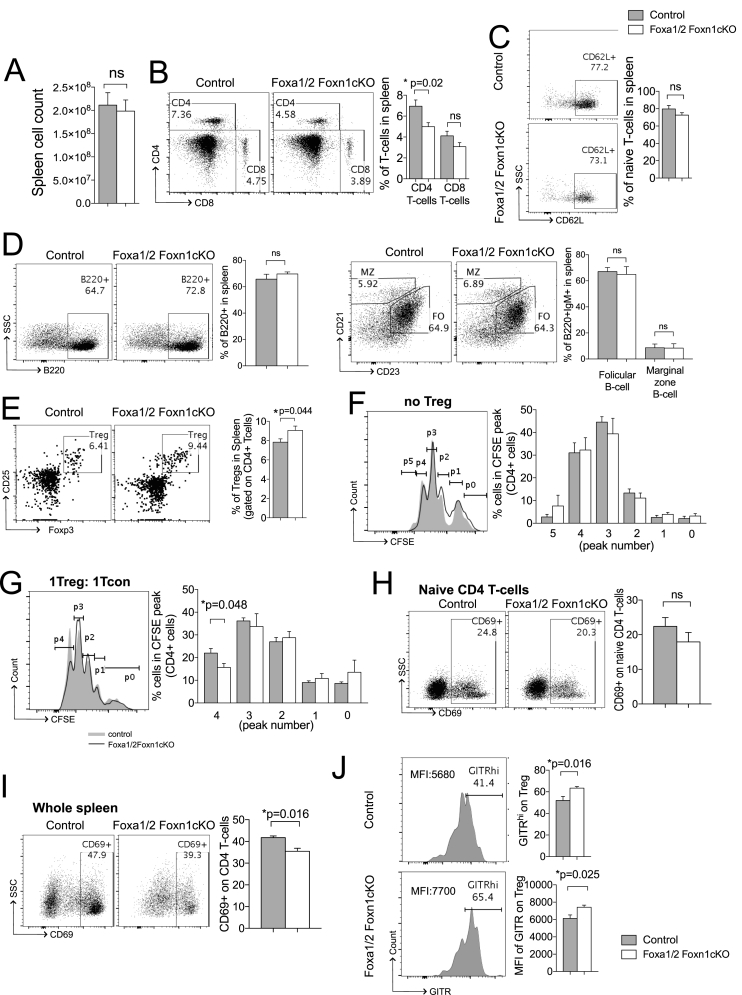
TEC-specific ablation of *Foxa1* and *Foxa2* impairs peripheral T-cell development. Flow cytometry analysis of spleen T-cells and B-cells. Unless otherwise stated data are from control (n = 5) and Foxa1/2Foxn1cKO (n = 5). (A) Bar chart shows mean ± SEM cell number of control and Foxa1/2Foxn1cKO spleen. (B) Dot plots show CD4 and CD8 expression. Bar chart shows mean ± SEM percentage of CD4 and CD8 T-cells in spleen. (C) Dot plots show CD62L expression, gated on CD4^**+**^CD44^**−**^ T-cells. Bar chart shows mean ± SEM percentage of CD4^**+**^CD44^**−**^CD62L^**+**^ (naïve) CD4 T-cells. (D, left panel) Dot plots show B220^**+**^ expression, giving percentage in region shown. Bar charts show mean ± SEM percentage B220^**+**^ splenocytes for control (n = 4) and Foxa1/2Foxn1cKO (n = 4). (D, right panel) Dot plots show CD21 versus CD23 expression, gated on B220^**+**^IgM^**+**^ population, follicular (FO) (CD21^**+**^CD23^**+**^) and marginal zone (MZ) (CD21^hi^CD23^**-**^) B-cells were identified. Bar chart shows mean ± SEM of percentage of follicular B-cells and marginal zone B-cell for control (n = 4) and Foxa1/2Foxn1cKO (n = 4) spleen. (E) Dot plots show staining of anti-CD25 against intracellular anti-Foxp3, gated on CD4 T-cells. Bar chart shows mean ± SEM percentage of Tregs in spleen. (F) Histograms show representative CFSE staining on control and Foxa1/2Foxn1cKO CD4 T-cells cultured for 4 days. (G) Histograms show representative CFSE staining on CD4 T-cells with control or Foxa1/2Foxn1cKO Tregs were added to Tcon at a 1:1 ratio for suppression assays. (F,G) The bar chart shows percentage of cells that have undergone the indicated numbers of cell divisions for control (n = 4) and Foxa1/2Foxn1cKO (n = 4). (H,I) Dot plots show facs profiles of CD69 staining on CD4^**+**^ T-cells in culture of whole spleen cell suspension (I) and FACS-sorted naïve CD4 T-cells (H), stimulated with anti-CD3 and anti-CD28 for 24 h. (J) Histograms show anti-GITR staining on CD4^**+**^CD25^**+**^Foxp3^**+**^ (Treg) cells, giving MFI. Bar chart shows mean ± SEM percentage of GITR^hi^ and MFI of anti-GITR for control (n = 4) and Foxa1/2Foxn1cKO (n = 4) spleen.

Analysis of the impact of conditional deletion of Foxa1 and Foxa2 from TEC on peripheral T-cell populations in the spleen showed that although the number of cells in spleen was similar between groups, the proportion of CD4 T-cells in the Foxa1/2Foxn1cKO spleen was significantly reduced compared to control (Fig. 5A–B). The proportion of naïve (CD62L^**+**^CD44^**−**^) cells in the CD4 population was not significantly different between genotypes, and we found no significant differences in the proportions of B-cells and marginal zone (CD21^hi^CD23^−^) and follicular (CD21^−^^+^CD23^+^) B cell populations (Fig. 5C–D). Interestingly, the CD4^+^CD25^+^Foxp3^+^Treg population was significantly increased in the Foxa1/2Foxn1cKO spleen compared to control (Fig. 5E). This increase in the Treg population may account for the reduction in CD4 T-cells in the spleen in Foxa1/2Foxn1cKO mice.

Therefore, to test this hypothesis and examine the possible mechanisms that might account for the reduction in the CD4 T-cell population in the Foxa1/2Foxn1cKO spleen, we measured the proliferation of activated CD4 T-cells from Foxa1/2Foxn1cKO and control spleen and compared the suppressive capacity of Tregs. We found there was no significant difference in cell proliferation between CD4 T-cells from control and Foxa1/2Foxn1cKO (Fig. 5F). In contrast, the proliferation of CD4 T-cells was significantly reduced in the presence of Foxa1/2Foxn1cKO Tregs at a 1:1 ratio (Treg cells:T conventional cells) compared to control Tregs (Fig. 5G), whereas at a ratio of 1:4 (Treg cells:T conventional cells) there was no significant difference in the level of suppression by Foxa1/2Foxn1cKO Tregs compared to control Tregs (data not shown).

To investigate activation of CD4 T-cells from Foxa1/2Foxn1cKO spleen, both total splenocytes and FACS-sorted naïve CD4 T-cells from control and Foxa1/2Foxn1cKO spleen were stimulated with anti-CD3/28 for 24 h and expression levels of the activation marker CD69 were assessed. Interestingly, there was no significant difference in CD69 expression between control and Foxa1/2Foxn1cKO CD4 T-cells when naïve T-cells were cultured and stimulated alone (Fig. 5H), whereas CD69 expression on CD4 T-cells was significantly decreased in Foxa1/2Foxn1cKO spleen compared to control when unfractionated spleen cell populations were cultured and stimulated with anti-CD3/28 (Fig. 5I). These results suggest that T-cell activation in Foxa1/2Foxn1cKO spleen was restricted only when APCs and Tregs were present in the culture. Analysis of expression of Glucocorticoid-induced tumour necrosis factor receptor-related protein (GITR), further confirmed that Foxa1/2Foxn1cKO Tregs had a more active phenotype than control Tregs, as both the MFI and percentage of GITR^+^ staining were significantly increased in the Foxa1/2Foxn1cKO Treg population (Fig. 5J).

Overall, these experiments suggest conditional deletion of Foxa1 and Foxa2 from TEC did not modify the cell-intrinsic proliferation of CD4 T-cells in the spleen, however, the suppressive activity of Tregs were enhanced and this most likely led to a reduction in the proportion of CD4 T-cells in the spleen and also affected CD4 T-cell activation.

In summary, our study shows that the pioneer transcription factors Foxa1 and Foxa2 regulate mTEC differentiation and the levels of cell surface MHCI and MHCII in TEC. Conditional deletion of *Foxa1* and *Foxa2* from TEC thus influenced thymocyte number, positive selection, CD4SP/CD8SP lineage commitment and Treg development in the thymus, and led to an increase in Tregs but reduction in CD4 T-cells in the spleen.

## 4 Discussion

The thymic microenvironment is essential for the development of T-cells, where the interaction between TEC and developing thymocytes supports T-cell development and governs the outcome of TCR repertoire selection. In this study, we presented a novel function for the transcription factors Foxa1 and Foxa2. We showed that their expression in TEC is required for normal TEC development and function, and thereby for the maintenance of T-cell development and homeostasis of thymic and spleen Treg populations.

Our data demonstrated that Foxa1 and Foxa2 are required for normal TEC differentiation and for physiological levels of cell-surface MHC expression on TEC. As conditional deletion of Foxa1 and Foxa2 from TEC led to reduced positive selection and differentiation to CD4 T-cell, we hypothesized that the reduction in the proportion of cTEC and in levels of MHCII cell-surface expression may influence the outcome of TCR repertoire selection by leading to fewer developing thymocytes being able to bind the MHCII + peptide ligands on cTEC, required for positive selection. Examination of the DP populations at the transition from DP to SP thymocyte, confirmed that fewer DP cells were entering the selection process in the Foxa1/2Foxn1cKO thymus, resulting in a reduction in the CD4SP population. Analysis of expression of CD5 and Nur77 showed that the TCR signal strength was not altered in thymocytes from the Foxa1/2Foxn1cKO thymus, thus indicating that the distorted positive selection was not the result of a thymocyte-intrinsic change in TCR signal strength.

mTEC play a key role in establishing self-tolerance, both by induction of clonal deletion of autoreactive T-cells and by supporting Treg differentiation. These processes require expression of Aire [39,40]. In human, Aire mutation results in loss of immune tolerance and development of autoimmune disease, and likewise in Aire^−^^/^^−^ mice tolerance is compromised and Treg populations are significantly reduced, leading to profound autoimmunity [41,42]. Our data showed that Foxa1 and Foxa2 are required for normal Aire^+^ mTEC differentiation. As a result of conditional deletion of Foxa1 and Foxa2 in TEC, Treg selection was increased in the thymus, and the proportion of Tregs were also increased in the Foxa1/2Foxn1cKO spleen. It would in the future be interesting to assess the consequences of increased Treg in Foxa1/2Foxn1cKO mice model on induction of autoimmunity and to investigate the clinical significance of the function of FOXA1 and FOXA2 in human TEC. A previous study has shown that Foxa1 is expressed in some T-cells and that Foxa1^**+**^CD4^**+**^ T-cells represent a distinct subset of Treg which play an immunosuppressive role in the central nervous system in a mouse model of multiple sclerosis [19]. Thus, the Foxa1 and Foxa2 transcription factors are important to our understanding of autoimmunity through their roles both in TEC and Treg function.

## Authorship contributions

Conceptualization: C-IL and TC; Investigation: C-IL, DCY, EP, AS, JIS, TC; Writing: C-IL and TC; Supervision, project administration and funding acquisition: TC.

## Disclosure of conflicts of interest

The authors report no conflict of interest.

## Appendix A Supplementary data

Supplementary Fig. 1 Histology Haematoxylin and Eosin staining of thymus (A-D), Spleen(E,F) and lymph node (G,H) sections from a pair of 3-wk-old control and Foxa1/2Foxn1cKO mice. Magnification and scale bar are indicated on the upper right and lower right of the pictures, respectively.
