# Supplementary figures and images for: Foxa1 and Foxa2 in thymic epithelial cells (TEC) regulate medullary TEC and regulatory T-cell maturation
Source: J Autoimmun. 2018 Sep;93:131–8. doi: 10.1016/j.jaut.2018.07.009 (PMC6119767; doi:10.1016/j.jaut.2018.07.009)

Supplementary Figure 1

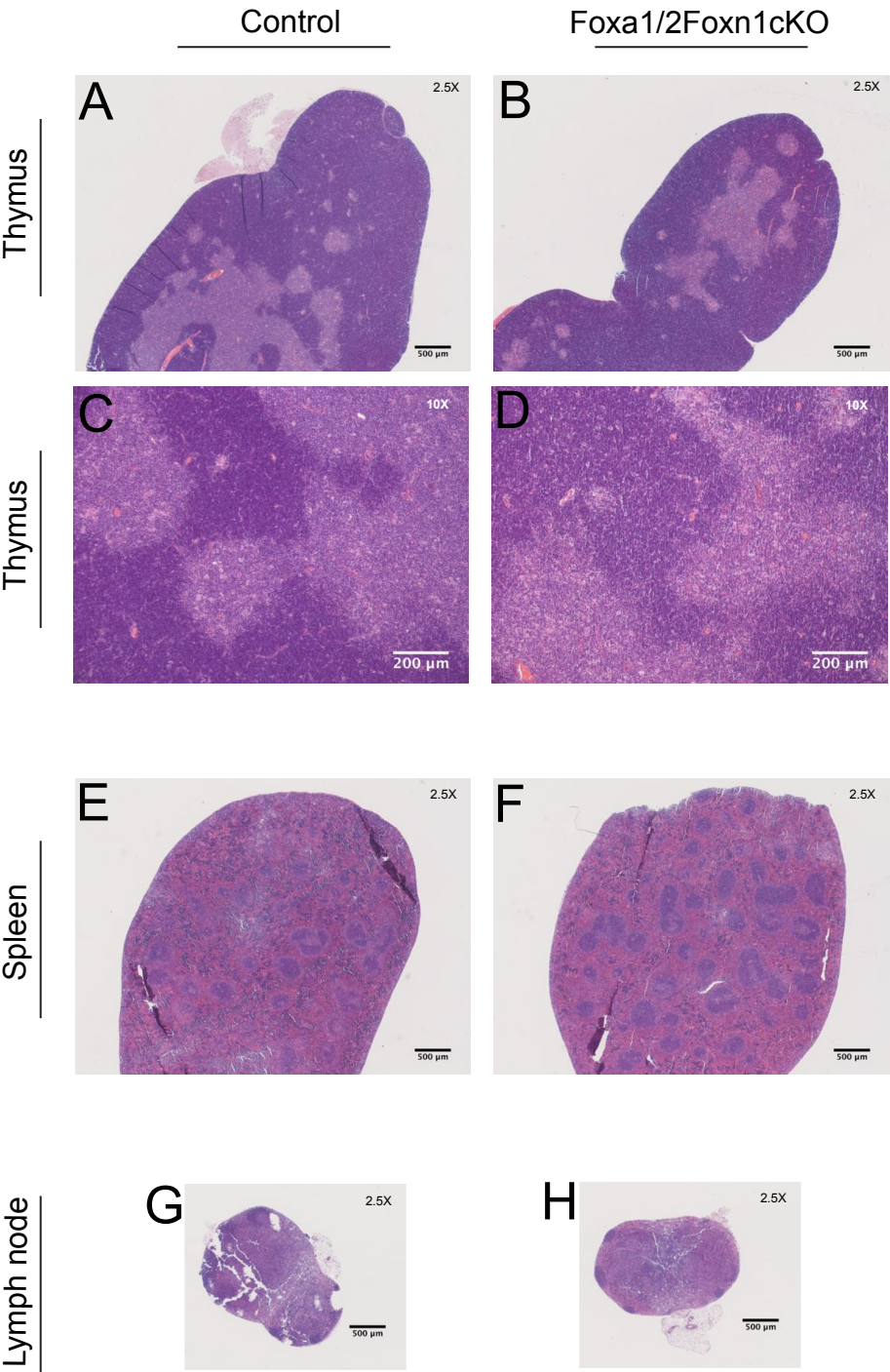

Supplement: Multimedia component 1 [file mmc1.pdf]
